# Current Immunotherapy Strategies and Emerging Biomarkers for the Treatment of Hepatocellular Carcinoma

**DOI:** 10.3390/cancers17233870

**Published:** 2025-12-02

**Authors:** Audrey Kapelanski-Lamoureux, Anthoula Lazaris, Nicholas Meti, Peter Metrakos

**Affiliations:** 1 Department of Anatomy and Cell Biology, McGill University, Quebec, QC H3A 0G4, Canada; audrey.kapelanski-lamoureux@mail.mcgill.ca; 2 Cancer Research Program, Research Institute of the McGill University Health Centre (RI-MUHC), Montreal, QC H3H 2R9, Canada; anthoula.lazaris@mail.mcgill.ca; 3 Cedars Cancer Center, Montreal, QC H4A 3J1, Canada; nicholas.meti@mcgill.ca; 4 McGill University Health Center (MUHC), Montreal, QC H4A 3J1, Canada; 5 Department of Surgery, Royal Victoria Hospital—McGill University Health Center, Montreal, QC H4A 3J1, Canada

**Keywords:** hepatocellular carcinoma (HCC), nonviral liver cancer, metabolically dysfunction-associated steatosis liver disease (MASLD), metabolic dysfunction-associated steatohepatitis (MASH), cancer biomarkers, diagnostic advancements, immunotherapy

## Abstract

Background/Objectives: Hepatocellular carcinoma (HCC), the predominant form of liver cancer, ranks as the third leading cause of cancer-related deaths worldwide. With the shift from viral hepatitis to metabolically dysfunction-associated steatosis liver disease (MASLD) and metabolic dysfunction-associated steatohepatitis (MASH) as primary etiologies, we aimed to review ongoing clinical trials in adult HCC patients to highlight emerging treatments, particularly for nonviral HCC cases. Methods: We searched ClinicalTrial.gov (last March 2025) for interventional trials. We included ongoing (recruiting/active/not recruiting), phase I-IV, adults (>18 years old), and HCC-focused only clinical trials. We excluded observational and interventional (biological, genetic, device, or procedure) clinical trials. Results: This review highlights recent advances in HCC treatment, with a focus on the transformative role of immunotherapy. Evidence suggests that nonviral HCC, as well as HCC with MASLD/MASH background livers, may have reduced sensitivity to immunotherapy. Thus, there is a critical need for molecular insights to improve patient stratification. Moreover, we examine how new diagnostic tools, including liquid biopsies, influence treatment decisions and aid in monitoring responses. Limitations limited MASLD/MASH-specific trial data. Conclusions: We review current research and its integration into clinical practice, advancing HCC therapy toward personalized, patient-centered care.

## Simple Summary

Hepatocellular carcinoma (HCC) remains one of the leading causes of cancer-related mortality worldwide. While viral hepatitis has historically been the predominant risk factor, the etiology of HCC is rapidly shifting, with metabolic dysfunction-associated steatosis liver disease (MASLD) and metabolic dysfunction-associated steatohepatitis (MASH) now emerging as the dominant etiologies. This shift has important clinical implications for therapy, as accumulating evidence suggests that MASLD/MASH-associated HCC is less responsive to immune checkpoint inhibitors.

## 1. Introduction

Liver cancer is a significant health issue worldwide, as it is the third most common cause of cancer-related death [1]. The most common liver cancer is hepatocellular carcinoma (HCC), accounting for about 75% to 95% of cases [1]. HCC is associated with cirrhosis in 80% of cases and is linked to various risk factors, including hepatitis B or C virus (HBV, HCV) and metabolic disease [2]. The implementation of hepatitis B vaccination and the development of curative treatments for HCV have led to a measurable decline in HBV- and HCV-related HCC cases in some countries; however, the global burden of HCC continues to rise [3,4,5]. This trend is partly associated with population growth and aging; however, a significant concern is the rising incidence of HCC associated with metabolic syndrome [3]. Approximately 38% of the global population suffers from metabolic dysfunction-associated steatosis liver disease (MASLD)*,* of which about 20% can progress to metabolic dysfunction-associated steatohepatitis (MASH), which represents a more severe form of the disease [6,7]. Of those with MASH, approximately 20% may progress to cirrhosis or end-stage liver disease [8]. MASLD is increasing with projections suggesting it may reach over 55% in adults by 2040, contributing to the rising incidence of HCC. Thus, the etiology of HCC is evolving, with MASH emerging as an increasingly important cause, particularly in Western countries [9].

HCC management follows a well-established staging system, the Barcelona Clinic Liver Cancer (BCLC) [10]. The BCLC guides therapeutic decisions for patients with HCC by integrating tumor burden, liver function, and patient performance status [10]. Curative strategies include surgical resection, liver transplantation, and local ablation, which are typically reserved for patients with early-stage (stage A) disease, while transarterial chemoembolization and systemic therapies, such as tyrosine kinase inhibitors (TKIs) and immune checkpoint inhibitors (ICIs), are more frequently administered in intermediate (stage B) to advanced (stage C) stages [10]. Despite these advances, recurrence remains a major clinical challenge, with up to 70% of patients experiencing relapse within five years after curative treatment [11,12]. This high recurrence rate underscores the urgent need for improving surveillance strategies for patients after curative interventions. Addressing these gaps is essential for optimizing surveillance, personalizing therapeutic interventions, and ultimately improving long-term outcomes.

A significant challenge in the field is the need for improved biomarkers for surveillance, as they are controversial [13]. The best-studied serological marker for HCC is Alpha-Fetoprotein (AFP) [14]. AFP is elevated in approximately 50–60% of HCC cases. Although AFP has been traditionally used as a biomarker for early-stage and recurrent HCC, recent studies indicate that AFP testing lacks both sensitivity and specificity [14,15]. AFP, in combination with ultrasound, remains a valuable tool for surveillance [16].

Additionally, imaging techniques such as MRIs and CT scans offer higher sensitivity [17]. However, their role as surveillance tools remains uncertain due to cost and availability [17]. As a result, many patients are diagnosed de novo with advanced and incurable disease, contributing to the poor prognosis with overall 5-year survival rates remaining as low as 22% for all stages combined [18,19], highlighting an unmet clinical need. The application of more modern molecular diagnostics is emerging as a critical tool in addressing the limitations of traditional methods for detecting HCC. Advances in technologies, such as liquid biopsies, such as circulating tumor DNA (ctDNA), circulating tumor cells (CTCs), and extracellular vesicles (EVs), are enabling the early detection of HCC [20,21]. These molecular tools offer a significant improvement over traditional biomarkers, such as AFP, as they provide high sensitivity and specificity for tumor detection [20,21]. These molecular diagnostic approaches are crucial for improving risk stratification, early detection, and recurrence strategies.

In addition to optimizing biomarker-based therapies for HCC, novel, more tailored systemic therapies are being actively explored. The introduction of immunotherapy demonstrated promising results in various clinical settings [22]. However, the distinct immunosuppressive tumor microenvironment of MASLD/MASH-associated HCC, characterized by reduced PD-L1 expression and T-cell receptor diversity, presents unique challenges to the efficacy of immunotherapy [23,24,25]. The objective of this review is to summarize the latest advances in molecular diagnosis and alternative treatment regimens for HCC, with a focus on nonviral and MASLD/MASH HCC.

## 2. Materials and Methods

We first conducted a literature review of ongoing interventional clinical trials for hepatocellular carcinoma (HCC) in adults, using publicly available data from ClinicalTrials.gov. This review describes the current pipeline, focusing on shifts toward nonviral etiologies (e.g., MASLD/MASH) and stratifying by intervention type. Inclusion criteria: Interventional trials (phase I–IV) registered on ClinicalTrials.gov; primary condition: hepatocellular carcinoma (HCC); adult participants (≥18 years); ongoing status (recruiting, active not recruiting, or not yet recruiting); primary completion date before 31 December 2024. Exclusion criteria: Observational studies; completed trials, pediatric trials (<18 years); non-HCC focused trials; non-interventional designs. Grouping for synthesis: Stratified by primary intervention: (1) immunotherapy (mono/combination), (2) targeted/systemic therapies, (3) treatment approaches, (4) MASLD/MASH-enriched and nonviral HCC subgroups. Results were tabulated: Table 1: Unresectable Hepatocellular Carcinoma First- and second-line therapy, Table 2: Resectable Hepatocellular Carcinoma immunotherapy for liver transplantation, Table 3: Resectable Hepatocellular Carcinoma Neoadjuvant Therapy, Table 4: Resectable Hepatocellular Carcinoma adjuvant therapy, Table 5: Resectable Hepatocellular Carcinoma peri-adjuvant therapy, Table 6: Unresectable Hepatocellular Carcinoma completed trials. Primary source: ClinicalTrials.gov (sole registry). Initial search: 15 January 2025; final update: 15 March 2025 (validated 27 October 2025).

## 3. Immunotherapy Treatment Pipeline for Unresectable HCC

### 3.1. First- and Second-Line Systemic Therapy Treatment

Immunotherapy in combination with anti-VEGF has become a cornerstone for the treatment of unresectable HCC, offering new therapeutic avenues to improve survival outcomes. Current guidelines highlight a stratified approach to systemic therapy for advanced (stage C) HCC, with immunotherapy-based regimens forming a central component of both first- and second-line treatment strategies (Table 1) [25,26]. In first-line therapy for unresectable HCC, patients are treated with a combination of atezolizumab (anti-PD-L1) and bevacizumab (anti-VEGF) [26]. Patients at higher risk of bleeding do not receive bevacizumab; instead, they generally receive combination immunotherapy with tremelimumab (anti-CTLA4) and durvalumab (anti-PD-L1) [27]. In situations where immunotherapy is unavailable, Lenvatinib (a multi-kinase inhibitor) is an alternative first-line treatment for unresectable HCC. According to the REFLECT trial, lenvatinib demonstrated noninferior efficacy to sorafenib, a tyrosine kinase inhibitor (TKI), in progression-free survival (PFS) [28,29]. In the event of lenvatinib intolerance or contraindication, sorafenib remains a viable alternative.

Patients who experience disease progression following first-line therapy can then access TKI therapy (i.e., lenvatinib or sorafenib) as second-line therapy, with sorafenib restricted to those with lenvatinib intolerance or a contraindication. If patients progress following TKI therapy, they are eligible for second-line TKI therapy, such as regorafenib or cabozantinib, which targets kinases similar to sorafenib and also has activity against VEGFRs, c-KIT, and TIE2. (Table 1) [25,26]. Regorafenib is supported by the RESORCE trial, which demonstrated an overall survival benefit for regorafenib in patients with HCC who had progressed on sorafenib [30]. Cabozantinib is supported by the CELESTIAL trial, which also demonstrated an overall survival benefit for cabozantinib in patients with advanced HCC who had received prior systemic therapy [31]. 

Systemic therapy treatment bases its guidelines on international evidence from pivotal phase III clinical trials, such as the use of atezolizumab plus bevacizumab or tremelimumab plus durvalumab in the first-line setting, which are aligned across international guidelines by the European Association for the Study of the Liver (EASL), the American Association for the Study of Liver Diseases (AASLD), Asia-Pacific Association for the Study of the Liver (APASL), and the Canadian Association for the Study of the Liver (CASL). However, significant differences exist in drug approval, public reimbursement, and accessibility, which can influence real-world treatment patterns [32].

### 3.2. Early Evidence Suggesting Reduced Efficacy of Immunotherapy in MASH-Associated HCC

Metabolically associated steatohepatitis (MASH) emerged as a leading risk factor of HCC and is associated with a distinct tumor microenvironment (TME), characterized by chronic inflammation and fibrosis [33,34,35]. Recent clinical trials and studies have suggested that TME may influence the response to ICIs. The following section summarizes mechanistic and foundational studies that fall outside the predefined clinical literature search but are included to provide essential biological context for interpreting etiological differences in HCC immunobiology.

Several immunosuppressive features characterize the MASH-associated TME. Initial preclinical data highlighting MASH’s immunosuppressive TME were provided by Pfiste et al., demonstrating how MASH can shape an immunosuppressive environment by impairing CD8+ T cell function [34]. Their work showed that MASH-induced HCC is characterized by T cell exhaustion, particularly the accumulation of exhausted CD8+ T cells with a CXCR6+ PD1 high phenotype [34]. This phenotype is indicative of auto-aggressiveness behavior and impaired liver immune surveillance, contributing to the immunosuppressive nature of the MASH-HCC TME [34]. In other studies, Mladenić, Karlo et al., and Apostolo, Daria et al. reported that in MASH, there is an increased presence of tumor-associated macrophages (TAMs) that exhibit immunosuppressive M2-like polarization [35]. These TMAs release anti-inflammatory cytokines and express immune checkpoint ligands, which can inhibit cytotoxic T cell function and contribute to resistance to ICIs [36,37]. The progression of MASH is also associated with increasing fibrosis, which further contributes to the immunosuppressive TME and acts as a physical barrier to immune cell infiltration [37]. Moreover, metabolic dysregulation can promote immune exhaustion by impairing T cell function [37]. These results underscore the importance of considering the underlying etiology of HCC when evaluating the efficacy of immunotherapy.

### 3.3. Immunotherapy Trials in HCC Highlight Differences in the Efficacy of ICIs Depending on the Underlying Etiology of HCC

As mentioned above, recent research suggests a potential difference in efficacy of ICIs depending on the underlying etiology of HCC [38]. While a direct comparison of viral-related HCC and/or MAFLD/MASH-related outcomes with ICIs has not been conducted, retrospective analyses and subgroup analyses provide insights into how HCC etiology may pose unique challenges to immunotherapy efficacy [38]. Below is a summary of key ICI phase III clinical trials and first- and second-line therapies, supporting the role of liver etiology in the efficacy of ICIs in HCC patients.

For first-line therapies, the current standard of care for unresectable HCC is based on the IMbrave150 trial. The trial demonstrated that atezolizumab plus bevacizumab significantly improved OS and PFS in patients with unresectable HCC [26,39]. While the trial showed clinical benefit in the overall studied population, subsequent subgroup analysis revealed nuanced findings. In patients with viral HCC (HBV or HCV), the subgroup analysis showed a trend toward a better outcome, suggesting that nonviral HCC may benefit less from immunotherapy. To further investigate the effectiveness of ICIs in nonviral HCC, a meta-analysis of prospectively collected data examined outcomes in patients with advanced nonviral HCC treated with atezolizumab plus bevacizumab, lenvatinib, or sorafenib [40]. The results showed that treatment with lenvatinib was associated with a longer OS [hazard ratio (HR) 0.65; 95% confidence interval (CI) 0.44–0.95; *p* = 0.0268] and PFS (HR 0.67; 95% CI 0.51–0.86; *p* = 0.002) compared to atezolizumab plus bevacizumab [40]. Thus, this suggests that patients with nonviral HCC may not benefit from immunotherapy compared with those with viral-related HCC, and that lenvatinib might be a more effective option for these patients. Notably, subgroup analysis of MAFLD/MASH-associated HCC was not reported in the IMbrave150 trial. However, in the same meta-analysis study, they examined MAFLD/MASH-related HCC and confirmed a longer OS (HR 0.46; 95% CI 0.26–0.84; *p* = 0.0110) and PFS (HR 0.55; 95% CI 0.38–0.82; *p* = 0.031) benefit with lenvatinib compared to atezolizumab plus bevacizumab [40]. Lastly, in the same study, the non-MAFLD/MASH-related HCC subgroup showed no differences in OS and PFS between the patients treated with lenvatinib and those treated with atezolizumab plus bevacizumab [40]. Thus, although IMbrave150 did not perform a MAFLD/MASH-related HCC subgroup analysis, these results align with the hypothesis that immunotherapy efficacy may vary by HCC etiology, with nonviral HCC and MAFLD/MASH-related HCC exhibiting attenuated responses compared with viral HCC.

Currently, there are no published, completed Phase III trials that directly compare atezolizumab plus bevacizumab to another immunotherapy for first- or second-line treatment of unresectable HCC. However, other completed Phase III clinical trials of HCC compare immunotherapy-based therapies with TKI-based therapies (e.g., HIMALAYA, LEAP-002, COSMIC-312, RATIONALE-301, ORIENT-32, CheckMate-9DW, CARES-310, and CheckMate-459) (Table 2). The HIMALAYA clinical trial is a phase III trial where a single priming dose of tremelimumab (anti-CTLA4) in combination with durvalumab (anti-PD-L1) against sorafenib in unresectable HCC [27]. The overall study population demonstrated superior OS compared to sorafenib (HR 0.78, 96.02% CI 0.65–0.93; *p* = 0.0035) [27]. Subgroup analysis did not present the more nuanced findings seen in the IMbrave150 trial.

In summary, the tremelimumab combination with durvalumab vs. sorafenib demonstrated a consistent OS benefit across various liver disease etiologies, including hepatitis B virus (HBV), hepatitis C virus (HCV), and nonviral causes [27]. In this trial, MAFLD/MASH-related HCC was not analyzed as a distinct etiological subgroup, although nonviral etiologies, including MAFLD/MASH, were included in the study population [27]. Overall, the HIMALAYA trials highlight that the benefit of immunotherapy-based treatments depends on the underlying etiology and the resulting tumor microenvironment. In the LEAP-002 trial, the lenvatinib plus pembrolizumab combination did not meet its primary endpoints of OS and progression-free survival (PFS) compared with lenvatinib plus placebo [41]. However, despite not reaching statistical significance for the primary endpoint, exploratory subgroup analyses showed that the combination was consistently favored across subgroups, including those with viral-related HCC aetiologies [41]. Similarly, the COSMIC-312 trial, comparing cabozantinib plus atezolizumab to sorafenib, demonstrated a significant reduction in the risk of disease progression or death but did not achieve a statistically significant improvement in OS [42]. Subgroup analyses revealed that the OS benefit from ICI therapies was more pronounced in the HBV subgroup than in the whole population. In contrast, no OS or PFS benefit was observed in patients with nonviral etiology [42]. In the RATIONALE-301 trial, comparing tislelizumab, an anti-PD-1, to sorafenib, tislelizumab demonstrated non-inferior OS, providing an alternative first-line treatment option [43]. While the trial met its non-inferiority primary endpoint, it did not show OS superiority over sorafenib. Subgroup analyses were conducted, and findings regarding viral versus nonviral etiologies were reported, indicating consistency across different etiologies [43]. In the ORIENT-32 trial, comparing sintilimab plus bevacizumab to sorafenib, the combination significantly improved OS and PFS [44]. The subgroup analyses confirmed a clear benefit in HBV-associated HCC (HR: 0.59 (95% CI 0.46–0.76)) for the combinational therapy, suggesting that this combination could provide a novel treatment option for such HBV-associated HCC. Lastly, in the CheckMate-459 trial, nivolumab (anti–PD-1) did not achieve statistical significance in improved OS compared to sorafenib [45]. Although exploratory subgroup analyses suggested that patients with viral-related hepatocellular carcinoma (HCC) might experience more favorable responses to immunotherapy than those with nonviral etiologies, these trends were not statistically significant within the trial itself. However, other analyses, including a meta-analysis that incorporates the CheckMate-459 data, have further explored the potential differences in outcomes between viral and nonviral HCC in the context of immunotherapy, showing more pronounced benefits for viral HCC [45]. As a result, although some trials observed differences in HCC responses by etiology, these differences were rarely statistically significant, suggesting that further study is necessary to determine conclusively whether viral-related HCC etiologies affect treatment efficacy. What could possibly confound the results is that the trials did not report whether the patients being treated for HCV had active viral disease. With further stratification, one could envision that the trends may become significant in those patients with active viral disease.

In the second-line setting, the KEYNOTE-240 phase III clinical trial evaluated pembrolizumab after sorafenib in advanced HCC, but it did not meet its prespecified statistical criteria for the co-primary endpoints of OS and PFS [46]. In subgroup analysis, patients with HBV etiology showed a clear OS benefit with pembrolizumab vs. placebo (HR: 0.57; 95% CI, 0.35–0.94), suggesting a more substantial treatment effect in this group. The analysis for the HCV subgroup showed an HR for OS of 0.96 (95% CI, 0.48–1.92), suggesting no apparent benefit of pembrolizumab over placebo in this group. In patients with nonviral, the HR for OS was 0.88 (95% CI 0.64–1.20) [46]. In the case of MAFLD/MASH-associated HCC patients, they were not analyzed explicitly in KEYNOTE-240 [46]. The KEYNOTE-240 trial, along with other studies, suggests a trend toward reduced immunotherapy benefits in nonviral HCC compared with viral HCC, providing additional support for the hypothesis that etiology influences treatment response. As a result of first- and second-line phase III clinical trials for advanced/unresectable HCC, we gained insights into how HCC etiology may need to be considered when designing ICI trials. While viral-related HCC (HBV and HCV) has been extensively studied, with dedicated subgroup analyses in most clinical trials, MAFLD/MASH-associated HCC has not been investigated as a distinct category in clinical trials. Instead, it is generally grouped among the nonviral HCC subgroup, despite accumulating evidence that MAFLD/MASH-related HCC should be considered as a separate category.

## 4. Biomarkers in HCC

To optimize patient selection for ICIs, significant research has focused on biomarkers within the tumor itself. Biomarkers such as PD-L1 expression, microsatellite instability (MSI), tumor mutational burden (TMB), and tumor-infiltrating lymphocyte (TIL) density have been extensively investigated as predictors of responsiveness to ICIs in HCC [47,48]. However, their clinical utility has been inconsistent. While PD-L1 expression is a commonly studied biomarker for ICI response, its predictive value in HCC is not uniformly established across clinical trials. For example, some trials, such as KEYNOTE-224 and CheckMate-459, demonstrated improved outcomes in PD-L1-positive HCC patients, whereas CheckMate-040 reported no significant correlation between PD-L1 levels and ICI efficacy. In contrast, the composition of the immune landscape appears more reliable. The tumor immune microenvironment plays a pivotal role in shaping therapeutic outcomes [49].

### 4.1. The Impact of Liver Disease Etiology on the Immune Landscape

Emerging evidence suggests that the underlying liver disease might significantly impact the therapeutic response to ICIs in patients with HCC [50]. Multiple clinical trials have demonstrated the efficacy of ICIs for HCC, but MAFLD/MASH-related HCC remains under investigation. The reason for this specific focus is the difference in tumor biology. MAFLD/MASH-related HCC appears to exhibit an immunosuppressive landscape with lower TIL infiltration compared to viral hepatitis-associated HCC. Recent biomarker studies have demonstrated that HCC associated with MAFLD/MASH typically shows reduced PD-L1 expression and lower T-cell receptor (TCR) diversity compared to viral HCC [9,23,51]. Additionally, recent studies have investigated the role of T-cell receptor (TCR) diversity in MAFLD/MASH-related HCC. Research shows that TCR diversity is reduced due to antigen-driven T-cell clonal expansion in MASH subpopulations, thereby impairing immune activity and response to immunotherapy [52,53]. These immunological features suggest that MASH-associated HCC may exhibit a reduced response to ICIs, underscoring the need to explore alternative therapeutic options, such as novel immunomodulators or combination therapies tailored to the unique biological characteristics of this patient subgroup [50].

### 4.2. Advancements in Molecular Diagnostics: Liquid Biopsy

Recent advancements in liquid biopsy technologies have introduced promising non-invasive biomarkers for the early detection and monitoring of disease. Extracellular vesicles (EVs), circulating tumor DNA (ctDNA), and circulating tumor cells (CTCs) offer new avenues for real-time tumor profiling [54]. Among these, EVs have gained increasing attention as potential biomarkers in HCC [55]. These small membrane-bound vesicles carry tumor-derived biomolecules such as proteins, RNAs, and DNA fragments, facilitating intercellular communication within the tumor microenvironment [55]. Importantly, their molecular cargo composition can reflect tumor progression and response to systemic therapy, making them valuable for early detection and treatment monitoring [56]. Specifically, EV-derived microRNAs (miRNAs) display disease-specific expression patterns in MAFLD/MASH-related HCC compared to other etiologies, highlighting their potential diagnostic specificity. MiR-21 and miR-122 are enriched in EVs, have been associated with tumor aggressiveness, and could serve as indicators of therapeutic response [57,58]. In addition, protein markers contained in EVs, such as glypican-3 and heat shock proteins, have been investigated for their diagnostic and prognostic relevance in HCC [59]. ctDNA analysis enables the detection of tumor-specific mutations, epigenetic alterations, such as methylation signatures and somatic mutations, and chromosomal alterations [60,61]. This approach provides a minimally invasive approach for assessing tumor burden, monitoring therapeutic efficacy, and tracking disease progression [61]. While ctDNA has been studied in HCC, studies specifically addressing its utility in MAFLD/MASH-related HCC are limited [60].

Despite these advances, traditional protein biomarkers remain important for HCC diagnosis. Alpha-fetoprotein (AFP) remains widely used but has limited sensitivity and specificity [62,63]. Glypican-3 (GPC3), in contrast, is highly overexpressed in HCC and has emerged as both a biomarker and a potential therapeutic target. In addition to its diagnostic role, GPC3 has become a focus for therapeutic innovation. Strategies such as GPC3-based vaccines and anti-GPC3 monoclonal antibodies are being actively explored as immunotherapeutic approaches in HCC [64,65,66,67,68]. Similarly, genetic predisposition has also been implicated in the development of MAFLD/MASH-related HCC. There is a strong association between the PNPLA3 I148M polymorphism and hepatic fat accumulation, fibrosis progression, and a heightened risk of HCC, making it a promising marker for risk-based screening in MAFLD/MASH-related liver disease [69].

### 4.3. Translating Liquid Biopsies into Clinical Practices

The potential of liquid biopsies in clinical practice extends beyond their diagnostic utilities. These technologies hold promise for early detection, treatment monitoring, and prognostication; however, significant challenges remain in their clinical implementation. Ensuring cost-effectiveness, accessibility, and validation across diverse patient populations will be critical to maximizing their impact on patient outcomes. In the clinical setting of early diagnosis, ctDNA analysis can detect HCC-specific genetic alterations before conventional imaging [70]. Similarly, EV-associated miRNAs and proteins offer promise as early biomarkers, potentially improving screening efficacy for MASH patients [71]. For treatment monitoring, serial ctDNA measurements can track tumor burden and detect minimal residual disease post-treatment [72]. Furthermore, dynamic changes in EV profiles may provide non-invasive insights into tumor progression or regression [73]. CTC enumeration, in parallel, may yield prognostic value by informing on metastatic potential and therapeutic responsiveness [74,75]. Despite these advances, translating liquid biopsy assays into routine clinical care requires large-scale validation, particularly in the context of MAFLD/MASH-related HCC. Prospective clinical trials are necessary to establish the predictive value and clinical utility of these approaches across heterogeneous patient populations. Equally significant, regulatory, technical, and economic barriers must be addressed to enable equitable adoption.

A significant obstacle to implementation is the lack of standardized operating procedures (SOPs) for assay performance, data interpretation, and clinical reporting. International organizations, such as the European Association for the Study of the Liver (EASL) and the American Association for the Study of Liver Diseases (AASLD), as well as regulatory agencies (e.g., FDA, EMA), are increasingly mandated to develop guidelines and centralized quality control frameworks [75]. These efforts are crucial for harmonizing testing across institutions and ensuring that validated assays are integrated into healthcare systems cost-effectively and sustainably.

As discussed above, biomarkers such as PD-L1, tumor mutational burden (TMB), microsatellite instability (MSI), and tumor-infiltrating lymphocyte (TIL) density can provide important information on the responsiveness to immune checkpoint inhibitors. Liquid biopsy technologies, including EVs, ctDNA, and CTCs, complement these tissue-based assays by enabling longitudinal tumor characteristics. Furthermore, genetic susceptibility markers, such as PNPLA3, may help in risk stratification and facilitate targeted surveillance strategies [76]. Looking forward, integrating multiomic approaches that combine genomic, transcriptomic, and epigenetic profiling promises to refine the utility of biomarkers and optimize therapeutic decision-making for patients with MAFLD/MASH-related HCC.

## 5. Treatment Strategies for HCC

Several staging systems are available that determine outcomes and treatment modalities. The Barcelona Cancer Liver Clinic (BCLC) [77], Italian Liver Cancer (ITA.LI.CA) [78], Hong Kong Liver Cancer (HKLC) [79], and Chinese Liver Cancer (CNLC) [80] are prominent staging and treatment algorithms used globally. The most widely used worldwide is the Barcelona Cancer Liver Clinic (BCLC) classification algorithm, initially established in 1999 and updated in 2022 and serving as a primary global reference [77].

The BCLC approach may be referred to as a “stage hierarchy”, whereby each disease stage is associated with a preferred treatment [81]. Treatment decisions are based on tumor characteristics, liver function, and overall patient health status [77]. Stages vary from very early (0), early (A), Intermediate (B), advanced (C), and terminal (D) [77]. The very early (0) and early (1) stages include patients with a single tumor of less than 5 cm or up to 3 nodules, each less than 3 cm each [77]. Treatment options include surgical resection if portal pressure and bilirubin are normal, liver transplantation if portal pressure/bilirubin levels are elevated with no significant comorbidities, or radiofrequency ablation (RFA) [77]. The intermediate (B) stage is for patients with multinodular who are asymptomatic and without vascular invasion or extrahepatic spread. The primary treatment option is transarterial chemoembolization (TACE) [77]. The advanced (C) stage includes patients with portal invasion, extrahepatic spread, and cancer symptoms. Treatment options include systemic therapy, with first-line options including the combination of atezolizumab-bevacizumab or durvalumab-tremelimumab, or single agents such as lenvatinib or sorafenib [77]. Second-line options include regorafenib, cabozantinib, or ramucirumab (for AFP-overexpressing HCC) [77]. The terminal (D) stage category is for severe liver dysfunction, and the recommended approach is best supportive care [77]. More recent guidelines introduce elements of treatment-stage migration or stage-specific alternatives, providing flexibility while maintaining a stage-hierarchy framework [81]. In parallel, emerging evidence, as summarized in this review, points to immunological differences between MASLD/MASH-related and viral-related HCC. Nevertheless, these differences have not yet translated into validated, etiology-specific treatment algorithms. Further research is needed to determine whether MASLD/MASH-related and viral-related HCC warrant divergent therapeutic strategies.

### 5.1. Neoadjuvant and Adjuvant Immunotherapy: Current Trials and Clinical Challenges

Despite setbacks with nivolumab (CheckMate-459) and pembrolizumab (Keynote-042) monotherapies, which did not achieve their primary endpoints in the advanced setting, immune checkpoint inhibitors remain a focus in HCC research [45,47,82]. However, it has shifted towards earlier stages of the disease, with systemic therapies now being investigated in the neoadjuvant and adjuvant settings to improve surgical outcomes and reduce recurrence rates, respectively [83]. These strategies are designed to leverage the immunomodulatory effects of checkpoint inhibitors, priming the immune system before surgery and maintaining immune surveillance afterward [84]. This summary provides an overview of current clinical trials, key findings, and challenges in this rapidly evolving field.

In the pre-operative neoadjuvant setting (Table 3), the objective is to shrink tumors, improve resectability, and potentially stimulate systemic immune responses [84]. Neoadjuvant strategies in HCC have gained interest due to their potential to improve R0 resection rates [84], reduce micrometastatic disease, and enhance long-term survival outcomes [84]. Several clinical trials have investigated various approaches, including systemic therapies such as tyrosine kinase inhibitors (TKIs) and immune checkpoint inhibitors (ICIs), as well as combination regimens, to evaluate their efficacy in the pre-operative setting [85]. Key metrics for assessing neoadjuvant efficacy include primary pathological response (MPR) and complete pathological response (pCR), which serve as surrogates for long-term prognosis, as well as objective radiological response and conversion-to-resectability rates [83,84,85,86]. Several clinical trials have evaluated the efficacy of neoadjuvant therapy in resectable HCC, each using distinct treatment regimens and outcome measures. The ongoing MORPHEUS-NEO HCC (NCT05908786) trial is a Phase Ib/II, open-label, multicenter, randomized platform study designed to assess the efficacy and safety of neoadjuvant immunotherapy combinations in patients with surgically resectable HCC. This trial investigates the combination of atezolizumab (anti-PD-L1), bevacizumab (anti-VEGF), tiragolumab (anti-TIGIT), and tobemstomig (bispecific CD3/PD-L1 antibody). The primary endpoint is MPR, while secondary outcomes include pCR, relapse-free survival (RFS), event-free survival (EFS), overall survival (OS), R0 resection rates, and Milan criteria downstaging, as well as safety and perioperative outcomes. In contrast, the NCT04721132 Phase II trial evaluates atezolizumab and bevacizumab as neoadjuvant therapy in patients with resectable HCC. The primary objective was to assess the safety and tolerability of this combinational therapy in the pre-operative setting. In contrast, the secondary objective includes evaluating the correlation between the rate of pathologic complete response, overall response rate at time of surgery (per Response Evaluation Criteria in Solid Tumors (RECIST 1.1 and modified RECIST 1.0)), duration of response as defined by time to recurrence/recurrence-free survival, in addition to overall survival. Additionally, NCT05807776 is a Phase II prospective study to evaluate the safety and efficacy of tislelizumab (anti-PD-1) monotherapy or in combination with lenvatinib as neoadjuvant therapy for resectable hepatocellular carcinoma. The primary endpoint is MPR (≥70% tumor necrosis), with monitoring of disease-free survival (DFS), objective response rate (ORR), microvascular invasion (MVI), surgical delay rates, and median OS (mOS). Collectively, these trials represent diverse neoadjuvant approaches that integrate immune checkpoint inhibitors, anti-angiogenic agents, and tyrosine kinase inhibitors to optimize surgical resectability, pathological response, and long-term patient outcomes in HCC.

An important challenge with neoadjuvant therapy is the lack of a standardized protocol for neoadjuvant immunotherapy in HCC. The optimal duration, combination, and patient selection remain unclear. Despite early and promising results, several clinical challenges remain for the adoption and optimization of immunotherapy in both neoadjuvant and adjuvant settings. These include optimizing treatment durations, managing immune-related adverse events, and identifying robust biomarkers for patient selection and treatment. A recent review discusses these in depth, emphasizing the necessity for further research [83,87].

In the context of adjuvant therapy (Table 4), the primary goal is to maintain immune activation post-surgery to prevent recurrence, which remains a major cause of mortality for patients with HCC [88]. To evaluate the efficacy of adjuvant strategies, clinical trials should focus on key outcomes such as recurrence-free survival (RFS), OS, and time to recurrence (TTR), as these endpoints directly measure the ability of adjuvant interventions to delay or prevent disease relapse [88]. The NCT05407519 is a multicenter, single-arm study evaluating the efficacy and safety of tislelizumab combined with sitravatinib (a TKI) as adjuvant therapy in HCC patients at high risk of recurrence after curative resection, with RFS and 2-year RFS as primary endpoints. Secondary outcomes include TTR, OS, OS rates at 12 and 24 months, and adverse events (AEs), assessing both efficacy and safety over a 24-month observation period. In contrast, the NCT03867084 (KEYNOTE-937) study will evaluate the safety and efficacy of pembrolizumab (MK-3475) versus placebo as adjuvant therapy in participants with HCC and a complete radiological response after surgical resection or local ablation, with RFS as the primary outcome and OS as a key secondary measure, monitored over 6–8 years. This trial also evaluates treatment-related adverse events (AEs), treatment discontinuation rates, and patient-reported quality of life (QoL) outcomes. Additionally, the NCT03383458 (CheckMate9DX) investigates whether nivolumab improves recurrence-free survival (RFS) compared to placebo in participants with HCC who have undergone complete resection or achieved a complete response after local ablation and are at high risk of recurrence. The primary endpoint is RFS, and secondary endpoints include OS and TTR, which are monitored over 49 months to 7 years. Currently, NCT05407519 is recruiting, with an estimated completion date of mid-2026. For KEYNOTE-937 and CheckMate9DX, the study is ongoing, and participants are receiving an intervention or being examined, but potential participants are not currently being recruited or enrolled. Their completion is estimated for the end of 2025. Collectively, these trials provide valuable insights into the role of immune checkpoint inhibitors in preventing recurrence and improving survival after resection, informing future strategies for adjuvant immunotherapy in HCC.

Lastly, peri-operative strategies (Table 5), which combine neoadjuvant and adjuvant interventions, are also being explored to maximize therapeutic efficacy [89]. Trial NCT04658147 evaluates nivolumab alone or combined with relatlimab, a LAG-3 inhibitor, in the neoadjuvant setting to help boost anti-tumor immunity. The AB-LATE-02 (NCT04727307), which studies the neoadjuvant atezolizumab and adjuvant atezolizumab plus bevacizumab in combination with percutaneous radiofrequency ablation of small HCC is a randomized phase II trial to reduce microscopic residual disease. NEOTOMA (NCT05440864) is a phase II, open-label, single-arm, multicenter study of tremelimumab in combination with durvalumab given prior to resection, followed by adjuvant durvalumab. At present, these peri-operative studies remain ongoing. For NCT04658147, participants are receiving an intervention or being examined, but potential participants are not currently being recruited or enrolled. Its completion is estimated for the end of 2026. The AB-LATE02 (NCT04727307) is still recruiting, with an estimated completion date in February 2031. Lastly, NEOTOMA (NCT05440864) is still recruiting, and it is estimated to be completed by the end of 2026. These trials underscore the growing recognition of the potential benefits of coordinated systemic interventions before and after surgery. 

### 5.2. Efficacy of Combinational Therapies

The success of atezolizumab and bevacizumab has increased interest in alternative combination strategies, especially in the neoadjuvant setting for resectable HCC. They have become increasingly popular due to their ability to target multiple pathways simultaneously, overcome resistance, and enhance overall efficacy. For HCC, ICIs, including atezolizumab, pembrolizumab, and nivolumab, are frequently combined with anti-angiogenic agents such as bevacizumab or lenvatinib [23,46]. These treatments aim to reduce angiogenesis, normalize tumor vasculature, and facilitate immune cell infiltration. Trial NCT04721132 combines atezolizumab and bevacizumab in the neoadjuvant setting to investigate their synergistic effects in reducing tumor burden and recurrence. Similarly, NCT04615143 (TALENT) evaluates tislelizumab in combination with lenvatinib for neoadjuvant treatment, aiming to enhance immune activation and inhibit angiogenesis. In the case of NCT05389527 (NeoLeap-HCC), it evaluates the efficacy and safety of neoadjuvant lenvatinib combined with pembrolizumab. Lastly, in the case of NCT05185739 (PRIMER-1), the efficacy of pre-operative pembrolizumab combined with lenvatinib compared to pembrolizumab and lenvatinib alone in terms of primary pathological response in patients with resectable HCC. This reflects the growing interest in expanding therapeutic targets beyond traditional angiogenesis and immune pathways.

The multimodal strategy typically involves combining targeted local therapy with systemic agents to achieve both local tumor control and systemic anti-tumor effects. NCT04857684 integrates the SBRT technology with atezolizumab and bevacizumab, leveraging the ability of SBRT to destroy localized tumor tissue while the systemic agents enhance immune responses and target residual microscopic disease. Similarly, NCT04727307 (AB-LATE02) incorporates radiofrequency ablation into systemic therapy regimens, utilizing ablation to eliminate localized tumors while systemic agents, such as atezolizumab and bevacizumab, promote immune surveillance. These approaches shed light on a shift toward personalized, comprehensive treatment paradigms that combine the strengths of local and systemic interventions to improve outcomes for patients with HCC.

### 5.3. Leveraging Molecular Insights for Tailored Treatment Plans

The use of genomic, transcriptomic, and proteomic profiling will play an essential role in identifying actionable biomarkers. This is exemplified in trials like MORPHEUS-NEO HCC (NCT05908786), which is designed with the flexibility to open new treatment arms as new agents become available, close existing treatment arms that demonstrate minimal clinical activity or unacceptable toxicity, or modify the participant population. Future clinical trials may incorporate adaptive designs that allow for real-time treatment modification based on molecular or imaging biomarkers. In addition to MORPHEUS-NEO HCC, the DYNAMIC (NCT04954339) trial assesses various endpoints, including pathologic response rates, relapse-free survival, and overall survival at multiple time points. By leveraging advances in precision oncology, researchers aim to improve patients’ survival in the ongoing clinical trials for resectable HCC.

## 6. Personalized Medicine in HCC

### 6.1. Integration of Molecular Diagnosis Treatment Approaches in HCC

Personalized medicine in HCC aims to not only optimize therapeutic outcomes and minimize unnecessary toxicities but also to leverage molecular and immunological insights for precise treatment selection [90]. Despite being the most widely adopted treatment algorithm for HCC worldwide, BCLC fails to fully address emerging molecular biomarkers and complex immunological mechanisms [91]. Ongoing clinical research and tumor profiling efforts are bridging these gaps, leading to a more refined and individualized treatment strategies that integrate targeted therapies and immunotherapies [91,92]. The molecular heterogeneity of HCC has led to the exploration of various targeted therapies involved in tumor growth and progression, including angiogenesis, immune checkpoint, Wnt/β-Catenin, and metabolic pathways [91]. Growing evidence indicates that distinct molecular subsets of HCC respond differently to immunotherapeutic agents, highlighting the need to incorporate biomarker-driven therapy selection [92]. Indeed, combination regimens pairing immunotherapy with small-molecule targeting agents against specific pathways hold promise for improving outcomes in advanced disease and will be explored below [26].

Angiogenesis plays a pivotal role in HCC progression as it promotes tumor vascularisation, and anti-angiogenic therapies such as lenvatinib, sorafenib, and bevacizumab aim to disrupt the tumor’s blood supply [28,93,94]. Two important clinical trials have demonstrated the impact of targeting this pathway on patient outcomes. The first one, IMbrave150 (NCT03434379), showed that atezolizumab combined with bevacizumab led to marked improvements in overall survival and progression-free survival compared to sorafenib in unresectable HCC, thus positioning it as a viable first-line therapy. The second one, REFLECT (NCT01761266), was non-inferior to sorafenib for OS while improving secondary endpoints such as progression-free survival and objective response rate, which became an alternative first-line option for patients with unresectable HCC. 

Immune checkpoint pathways are also essential to understanding and treatment of HCC, as they regulate how tumors can modulate the immune system. These clinical trials highlight the growing interest in targeting novel immune checkpoint and co-stimulatory pathways in HCC. Some phase III trials, such as HIMALAYA, focus on combining ICIs that have shown efficacy individually, while others, like KEYNOTE-240 and CheckMate 040, evaluate single-agent ICIs in advanced HCC [95]. The NCT04658147 is an ongoing phase II clinical trial for therapy-related relatlimab (anti-LAG-3) with or without nivolumab (anti-PD-1), aiming to determine the safety and tolerability of the neoadjuvant/adjuvant combination for patients with potentially resectable HCC.

The Wnt/β-Catenin pathway is frequently dysregulated in HCC and is a primary driver of tumor growth and resistance to immunotherapy [96,97]. The Wnt/β-Catenin pathway targets are under ongoing investigation using small-molecule inhibitors. Early investigations include clinical trials NCT01351103, which examined LGK974 as a Wnt pathway inhibitor in malignancies dependent on this pathway, including HCC, and NCT02069145, which studied OMP-54F28 in combination with sorafenib in patients with HCC.

In the case of metabolic pathways, they also play a significant role in HCC, particularly in the context of MASH, where the dysregulation of lipid and glucose metabolism contributes to tumor progression [34]. To date, no dedicated, stand-alone clinical trial has evaluated lipid-synthesis pathway modulation as a direct therapy for HCC. However, it is hypothesized that normalizing metabolic pathways in patients at risk could reduce progression to HCC. For example, the FASCINATE-1 phase IIb clinical trial (NCT04906421) is investigating TVB-2640, an oral fatty acid synthase (FASN) inhibitor, to assess its safety and efficacy in patients with MASH. Although this clinical trial focuses on MASH rather than HCC, it would be important to track HCC risk in these patients because advanced MASH and cirrhosis carry an elevated HCC risk. 

Looking ahead, the incorporation of platforms such as next-generation sequencing (NGS) and liquid biopsies to enable the identification of actionable driver mutations and real-time monitoring of minimal residual disease [54,98]. These advancements, together with robust clinical trial data supporting the use of targeted therapies, will help propel the development of individualized treatment strategies [91]. Continued exploration of these pathways in combination with systemic immunotherapies or localized treatments will further refine personalized treatment approaches.

### 6.2. Implications of Liver Transplant

Liver transplant (LT) is considered the best curative treatment for select patients with HCC, particularly those meeting the Milan criteria (a single tumor ≤5 cm or up to three tumors ≤3 cm each, without vascular invasion or distant metastasis) [99]. By replacing the diseased liver, LT addresses both the tumor and the underlying liver disease [100]. For patients with HCC who do not respond to systemic therapies or have contraindications to them, LT may serve as a curative option if they meet transplant eligibility criteria [101].

The current LT algorithm adheres to established criteria from the Milan criteria and the University of California, San Francisco (UCSF) criteria [102,103]. The potential integration of LT into personalized HCC management strategies highlights the importance of considering patients who are unresponsive to systemic therapies’ eligibility for transplant [104]. However, concerns remain about the safety and timing of ICIs in the pre- and post-LT settings [105]. In the pre-LT setting, ICIs may increase the risk of immune-related adverse events (irAEs), including graft rejection in the post-LT setting [105]. The use of ICIs in the post-transplant setting may be counteracted by immunosuppressive therapy to prevent graft rejection, but may also cause organ rejection [106]. 

However, early data suggest that careful patient selection and management may mitigate these risks, enabling the safe integration of ICIs into transplant pathways [106] (Table 6). Clinical trial NCT05027425 investigates the use of ICI before LT in patients with HCC to determine whether ICIs can improve pre-treatment disease management without increasing the risk of graft rejection. Clinical trial NCT05185505 focuses on combining ICIs with loco-regional treatment before LT to optimize tumor control while patients wait for transplantation, potentially increasing transplant success rates. Lastly, the NCT05355155 clinical trial investigates the outcomes of LT in patients with HCC who have previously received ICIs, aiming to establish protocols for integrating ICIs into transplant pathways while addressing concerns about immune-related adverse events (irAEs) and graft rejection [105]. The NCT05027425 study is currently ongoing, with participants receiving an intervention or undergoing examination. However, potential participants are not being recruited or enrolled at this time. This study is estimated to end in 2030. The NCT05185505 trial remains open for recruitment and is expected to conclude in 2027. Finally, NCT05355155 has been completed, but results have not yet been posted.

In the case of MAFLD/MASH-related HCCs, LT is a compelling option, as this population often shows limited responsiveness to immunotherapy [34]. Expanding the current transplant algorithm to include patients with immunotherapy-resistant HCC could help ensure equitable access to curative treatments. However, further research is needed to establish the safety and efficacy of this approach, particularly in the context of immunotherapy resistance, a unique challenge posed by MAFLD/MASH-related HCC [106].

## 7. Conclusions

HCC, particularly in a nonviral context such as MASH, poses significant treatment challenges. The introduction of ICIs into first- and second-line therapies, as shown by the IMbrave150 trial combining atezolizumab–bevacizumab and the HIMALAYA trial combining tremelimumab–durvalumab, has redefined therapy for unresectable HCC. However, subgroup analyses reveal attenuated benefit in nonviral and MAFLD/MASH-related HCC, mirroring preclinical evidence that steatohepatitis fosters an immunosuppressive tumor microenvironment as discussed in this review. Advances in molecular diagnostics and biomarker development are central to precision oncology in HCC. Classical markers provide prognostic and predictive insights, while liquid biopsy platforms enable minimally invasive real-time monitoring. Emerging data in MAFLD/MASH-related conditions underscore the need for etiology-tailored biomarkers. Therapeutic strategies are evolving towards multimodal, molecularly guided therapies. Neoadjuvant and adjuvant immunotherapy trials aim to increase resectability and decrease recurrence, while combining ICI strategies aim to overcome mechanisms of treatment resistance. Concurrently, artificial intelligence-driven decision-making integrates imaging, molecular profiling, and clinical parameters to optimize individualized treatment pathways and adaptive trial design (not covered in this review). Therefore, future progress relies on etiology-specific clinical trials, the development of biomarkers for MAFLD/MASH-related HCC cohorts, the implementation of multi-omic profiling for treatment selection, and the use of artificial intelligence-based analytics for dynamic decision-making. Together, these efforts establish a precision-guided oncology framework for HCC, particularly for MAFLD/MASH-related HCC.

## Figures and Tables

**Table 1:** Unresectable Hepatocellular Carcinoma First- and second-line therapy.

First Line	Second Line
Atezolizumab + Bevacizumab	Regorafenib
Tremelimumab + Durvalumab (STRIDE regimen)	Carbozantinib
Nivolumab + Ipilimumab	Ramucirumab (for AFP ≥ 400 ng/mL)
Lenvatinib	
Sorafenib	

This table summarizes the systemic therapy options for unresectable hepatocellular carcinoma. First-line treatments include atezolizumab–bevacizumab, tremelimumab–durvalumab, nivolumab–ipilimumab (where approved), lenvatinib, and sorafenib. Second-line therapies consist of regorafenib, cabozantinib, and ramucirumab, all validated in patients previously treated with sorafenib.

**Table 2:** Resectable Hepatocellular Carcinoma immunotherapy for liver transplantation.

NCT Number	Phases	Interventions	Primary Outcome	Secondary Outcome *	Start Date
NCT06327269	EARLY_PHASE1	Lenvatinib	RFS	None	1 April 2021
NCT06337162	PHASE1	INCB099280	Acute Cellular Rejection of Liver Transplant Attributed to Study Therapy	Acute Cellular Rejection of Liver Transplant Within 3 months	1 May 2024
NCT05355155	PHASE2	Bevacizumab Biosimilar IBI305 + FOLFOX4	ORR based on RECIST 1.1	PFS	1 May 2022
NCT05027425	PHASE2	Durvalumab + Tremelimumab + Liver Transplant	Cellular rejection rates	Adverse events during treatment, and graft loss and mortality rates	7 December 2021
NCT05475613	PHASE2	Neoadjuvant/downstaging treatment containing Anti-PD-1 inhibitor (Tislelizumab, Pembrolizumab, Nivolumab et al.) followed by liver transplant if downstaging was successful	For patients with liver transplantation: The 2-year event-free survival rate	For patients with liver transplantation: The 2-year overall survival. For all patients with HCC downstaging: 1, 2 year overall survival rate	1 August 2023
NCT05576909	PHASE2	Neoadjuvant/downstaging treatment containing TACE and adjuvant Donafenib	Downstaging success rate	ORR before transplantation	30 March 2023
NCT05475613	PHASE2	Neoadjuvant/downstaging procedures containing Anti-PD-1 inhibitor (Tislelizumab, Pembrolizumab, Nivolumab et al.) followed by liver transplant if downstaging was successful	For patients with liver transplantation: The 2-year event-free survival rate	For patients with liver transplantation: The 2-year OS. For all patients with HCC downstaging: 1, 2 year OS	1 August 2023
NCT06041490	PHASE2	Multi-kinase inhibitors (Sorafenib or Lenvatinib or Donafenib) + Bevacizumab vs. No Intervention: Without adjuvant therapy	RFS (1-year)	RFS	1 September 2023
NCT05185505	PHASE4	Neoadjuvant/downstaging Atezolizumab + Bevacizumab + TACE	Proportion of Patients Receiving Liver Transplant Experiencing Acute Rejection	Proportion of participants who experience treatment-emergent adverse events	30 January 2023

The table summarizes clinical trials on the use of immune checkpoint inhibitors (ICIs) as adjuvant therapy following liver transplantation for patients with hepatocellular carcinoma. Abbreviations: FOLFOX4; Folinic acid (leucovorin), Fluorouracil (5-FU), and Oxaliplatin; PD-1, Programmed Cell Death Protein 1; TACE, Transarterial Chemoembolization; RFS, Recurrence-Free Survival; ORR, Objective Response Rate; RECIST, Response Evaluation Criteria in Solid Tumors; PFS, Progression-Free Survival; OS, Overall Survival. * Only one secondary outcome measure is presented for brevity. Additional secondary outcome measures were reported in the original trial publication.

**Table 3:** Resectable Hepatocellular Carcinoma Neoadjuvant therapy.

NCT Number	Phases	Interventions	Primary Outcome	Secondary Outcome *	Start Date	Completion Date
NCT04857684	EARLY PHASE1	SBRT + Atezolizumab + Bevacizumab	TRAE assessed by CTCAE	ORR assessed by RECIST 1.1	18 June 2021	31 December 2025
NCT04224480	PHASE1	Pembrolizumab	RR	None	10 December 2019	31 December 2025
NCT05225116	PHASE1	Sintilimab + Lenvatinib + Radiotherapy	AEs assessed by CTCAE	MPR	8 January 2023	5 December 2025
NCT05185531	PHASE1	Tislelizumab + SBRT	Delay to surgery, ORR	DFS	1 March 2022	1 December 2024
NCT05908786	PHASE1|PHASE2	Atezolizumab + Bevacizumab vs. Atezolizumab + Bevacizumab + Tiragolumab vs. Bevacizumab + Tobemstomig	MPR	PCR	5 December 2023	30 September 2028
NCT06492408	PHASE2	Double ICIs combination: Ipilimumab +Pembrolizumab or Ipilimumab + Durvalumab, Ipilimumab + Pembrolizumab or Ipilimumab + Durvalumab combined with Idarubicin, Ipilimumab + Pembrolizumab or Ipilimumab + Durvalumab combined with Idarubicin plus Bevacizumab vs. double ICIs + Idarubicin vs. Double ICIs + Idarubicin + Bevacizumab	pCR	Toxicity assessed by CTCAE	1 July 2024	30 December 2033
NCT04721132	PHASE2	Atezolizumab + Bevacizumab	pCR	ORR	10 February 2021	31 December 2027
NCT06420440	PHASE2	HAIC + Lenvatinib + Tislelizumab followed with liver resection with adjuvant Tislelizumab vs. Liver resection with adjuvant Tislelizumab	EFS	Safety assessed by CTCAE	1 June 2024	31 May 2027
NCT06512467	PHASE2	Donafenib combined with Sintilimab + HAIC	MPR	ORR	30 July 2024	31 December 2026
NCT06467799	PHASE2	FOLFOX-HAIC combined with tislelizumab, followed by surgical resection + tislelizumab	RFS	ORR	1 July 2024	1 July 2026
NCT06405061	PHASE2	Adebrelimab	ORR	MPR	1 May 2024	1 August 2027
NCT06349317	PHASE2	IMRT combined with Camrelizumab + Apatinib	EFS (1 year)	EFS	22 April 2024	1 June 2026
NCT05920863	PHASE2	Lenvatinib Combined with Tislelizumab + TACE	MPR	pCR	1 July 2023	1 March 2026
NCT05807776	PHASE2	Tislelizumab vs. Tislelizumab + Lenvatinib	MPR	DFS (1-year and 2-years)	1 April 2023	31 December 2025
NCT05389527	PHASE2	Pembrolizumab + Lenvatinib	MPR	pCR	30 September 2022	31 July 2025
NCT05578430	PHASE2	Cadonilimab + TACE followed by surgery	MPR	1-year recurrence rate	1 January 2023	1 January 2025
NCT04850040	PHASE2	Camrelizumab in Combination with Apatinib and Oxaliplatin	MPR	ORR	6 May 2021	31 December 2024
NCT05194293	PHASE2	Durvalumab + Regorafenib	ORR	Rate of surgery	1 July 2023	5 December 2028
NCT06311916	PHASE4	HAIC + Tirelizumab + lenvatinib followed by liver resection	DFS	Safety	1 May 2024	31 December 2028
NCT05660213	PHASE4	Targeted therapies: Atezolizumab + Bevacizumab or Camrelizumab + Apatinib or Sintilimab + Bevacizumab vs. TACE combined with targeted therapies vs. Lenvatinib vs. Huaier granule combined with any of the above regimens	ORR	PFS	30 January 2024	1 January 2027

The table summarizes clinical trials on the use of immune checkpoint inhibitors (ICIs) as neoadjuvant therapy in resectable hepatocellular carcinoma. Abbreviations: ORR, Objective Response Rate; MPR, Major Pathologic Response; DFS, Disease-Free Survival; pCR, Pathologic Complete Response; CTCAE, Common Terminology Criteria for Adverse Events; EFS, Event-Free Survival; PFS, Progression-Free Survival; RFS, Recurrence-Free Survival; SBRT, Stereotactic Body Radiation Therapy; ICI, Immune Checkpoint Inhibitors; HAIC, Hepatic Arterial Infusion Chemotherapy; FOLFOX, Folinic acid (leucovorin), Fluorouracil (5-FU), and Oxaliplatin; IMRT, Intensity-Modulated Radiotherapy; TACE, Transarterial Chemoembolization. * Only one secondary outcome measure is presented for brevity. Additional secondary outcome measures were reported in the original trial publication.

**Table 4:** Resectable Hepatocellular Carcinoma adjuvant therapy.

NCT Number	Phases	Interventions	Primary Outcome	Secondary Outcome *	Start Date	Completion Date
NCT05701488	PHASE1	Durvalumab + Tremelimumab vs. Durvalumab + Tremelimumab + SIRT	AE by CTCAEv5	Best Radiologic Response	21 April 2023	1 October 2025
NCT06454578	PHASE2	Adebrelimab + Apatinib	RFS	OS	24 July 2024	1 June 2027
NCT06498622	PHASE2	Donafenib + Envafolimab	RFS	OS	20 July 2024	20 May 2027
NCT05516628	PHASE2	Atezolizumab-Bevacizumab	RFS	TTR	28 February 2023	1 March 2027
NCT06143579	PHASE2	FOLFOX-HAIC+Lenvatinib+Envolizumab	AEs	pCR	15 December 2023	31 December 2026
NCT06546280	PHASE2	Camrelizumab + Apatinib	RFS (1-year)	RFS	26 August 2024	1 September 2026
NCT05407519	PHASE2	Tislelizumab + Sitravatinib	RFS (2-years)	TTR	25 July 2022	30 June 2026
NCT05367687	PHASE2	Camrelizumab + Rivoceranib vs. Camrelizumab	RFS determined by the investigator	RFS (24, 36 months) assessed by the investigator	1 September 2022	30 April 2026
NCT05389527	PHASE2	Pembrolizumab + Lenvatinib	MPR	pCR	30 September 2022	31 July 2025
NCT04981665	PHASE2	TACE followed by Tislelizumab	RFS (2-years)	RFS	8 November 2021	1 December 2024
NCT03867084	PHASE3	Pembrolizumab vs. Placebo	RFS assessed by BICR, overall survival	AE	28 May 2019	31 August 2029
NCT04102098	PHASE3	Atezolizumab + Bevacizumab	RFS	OS	31 December 2019	16 July 2027
NCT03847428	PHASE3	Durvalumab ± Bevacizumab vs. Placebo	RFS per RECIST 1.1 criteria as assessed by BICR	RFS	29 April 2019	31 May 2027
NCT03383458	PHASE3	Nivolumab vs. Placebo	RFS	OS	18 April 2018	16 December 2025
NCT04682210	PHASE3	Sintilimab + Bevacizumab vs. Active Surveillance	RFS	OS	1 December 2020	1 December 2024
NCT06311929	PHASE4	PD-1 monoclonal antibody and Lenvatinib vs. PD-1 monoclonal antibody	DFS	OS	1 April 2024	31 December 2028
NCT06496815	PHASE4	Donafenib vs. Donafenib + TACE or HAIC + PD-1, PD-L1	ORR according to mRECIST, PFS	DCR	1 August 2024	30 July 2027

The table summarizes clinical trials on the use of immune checkpoint inhibitors (ICIs) as adjuvant therapy in resectable hepatocellular carcinoma. Abbreviations: AE, Adverse Event; CTCAE, Common Terminology Criteria for Adverse Events; RFS, Recurrence-Free Survival; MPR, Major pathologic response; BICR, Blinded Independent Central Review; DFS, Disease-Free Survival; ORR, Objective Response Rate; OS, Overall Survival; TTR, Time to Recurrence; pCR, Pathologic Complete Response; mRECIST, modified Response Evaluation Criteria in Solid Tumors; RECIST, Response Evaluation Criteria in Solid Tumors; TACE, Transarterial Chemoembolization; HAIC, Hepatic Arterial Infusion Chemotherapy; DP-1, Programmed Cell Death Protein 1; PD-L1, Programmed Death-Ligand 1. * Only one secondary outcome measure is presented for brevity. Additional secondary outcome measures were reported in the original trial publication.

**Table 5:** Resectable Hepatocellular Carcinoma peri-adjuvant therapy.

NCT Number	Phases	Interventions	Primary Outcome	Secondary Outcome *	Start Date	Completion Date
NCT04658147	PHASE1	Nivolumab vs. Nivolumab + Relatlimab	Number of patients who complete pre-op treatment and proceed to surgery, 4 years	Toxicity as defined by CTCAE	28 May 2021	1 June 2026
NCT04727307	PHASE2	Neoadjuvant Atezolizumab + RFA and adjuvant Atezolizumab + Bevacizumab vs. Neoadjuvant Atezolizumab and adjuvant Atezolizumab + Bevacizumab + RFA	RFS	None	26 January 2021	1 February 2031
NCT05440864	PHASE2	Durvalumab + Tremelimumab, followed by surgery and adjuvant Durvalumab	Number of greater than grade 3 AEs	Number of patients who experience a surgical delay due to TRAEs	26 October 2023	1 November 2026
NCT06031285	PHASE2	Sintilimab + Bevacizumab Biosimilar + TACE-HAIC	RFS		1 September 2023	30 December 2025
NCT05519410	PHASE2	Sintilimab + Lenvatinib vs. HAIC-FOLFOX	DFS (1-year)	ORR	23 August 2022	1 September 2025
NCT04954339	PHASE2	Aatezolizumab + Bevacizumab	pCR, rate of dynamic changes using single nucelar RNA-sequencing, single cell RNA sequencing, spatial transcriptomics, multiplexed immunohistochemistry (mIHC), flow cytometry (and/or CyTOF)	The rate of completion of treatment and resection	29 October 2021	31 December 2026
NCT05613478	PHASE3	TACE + Camrelizumab + Apatinib mesylate followed by resection and adjuvant Camrelizumab and Apatinib mesylate vs. adjuvant TACE, and adjuvant Camrelizumab + Rivoceranib	RFS	MPR	1 November 2022	1 November 2027
NCT03916627	PHASE2	Cemiplimab followed by surgery and adjuvant Cemiplimab and Platinum doublet vs. Cemiplimab and Platinum doublet followed by surgery and adjuvant Cemiplimab and Platinum doublet vs. Platinum doublet followed by surgery Cemiplimab and Platinum doublet	MPR, STN, MTE	Delay to surgery, defined as surgery >28 days following the end of the second cycle of cohort-specific neoadjuvant therapy	23 July 2019	26 February 2031
NCT05185739	PHASE2	Pembrolizumab + Lenvatinib vs. Pembrolizumab vs. Lenvatinib	MPR	Percentage of viable tumor cells at resection	25 August 2022	1 July 2026
NCT04615143	PHASE2	Tislelizumab vs. Tislelizumab + Lenvatinib	DFS	ORR	1 December 2020	1 December 2025
NCT04912765	PHASE2	Neoantigen Dendritic Cell Vaccine + Nivolumab	RFS (24-month)	AEs	15 April 2021	1 May 2025

The table summarizes clinical trials on the use of immune checkpoint inhibitors (ICIs) as peri-adjuvant therapy in resectable hepatocellular carcinoma. Abbreviations: RFA, Radiofrequency Ablation; AE, Adverse Events; RFS, Recurrence-Free Survival; DFS, Disease-Free Survival; MTE, Major treatment effect; STN, Significant tumor necrosis; MPR, Major pathologic response; RFS, Recurrence-Free Survival; ORR, Objective Response Rate; TRAE, Treatment Related Adverse Events; TACE, Transarterial Chemoembolization; HAIC, Hepatic Arterial Infusion Chemotherapy; FOLFOX, Folinic acid (leucovorin), Fluorouracil (5-FU), and Oxaliplatin; CTCAE, Common Terminology Criteria for Adverse Events. * Only one secondary outcome measure is presented for brevity. Additional secondary outcome measures were reported in the original trial publication.

**Table 6:** Unresectable Hepatocellular Carcinoma completed trials.

NCT Number	Phases	Interventions	Treatment	Primary Outcome	Secondary Outcome *	Start Date	Completion Date
NCT03794440 (ORIENT-32)	PHASE2|PHASE3	Systemic	Sintilimab + IBI305 vs. Sorafenib	OS, PFS	PFS	11 February 2019	1 December 2022
NCT03434379 (IMBRAVE150)	PHASE3	Systemic	Atezolizumab + Bevacizumab vs. Sorafenib	OS, by PFS-IRF per RECIST v1.1	ORR	15 March 2018	17 November 2022
NCT03755791 (COSMIC-312)	PHASE3	Systemic	Cabozantinib + Atezolizumab vs. Cabozantinib vs. Sorafenib	PFS, OS	PFS per RECIST 1.1 by BIRC	10 June 2018	1 December 2024
NCT03764293 (CARES-310)	PHASE3	Systemic	SHR-1210 + Apatinib vs. Sorafenib	OS, PFS per BIRC based on RECIST v1.1	ORR	10 June 2019	14 June 2023
NCT03713593 (LEAP-002)	PHASE3	Systemic	Lenvatinib + Pembrolizumab vs. Lenvatinib + Saline placebo	PFS as determined by BICR per RECIST 1.1 or death due to any cause	ORR	31 December 2018	24 September 2024
NCT03298451 (HIMALAYA)	PHASE3	Systemic	Durvalumab or Durvalumab + Tremelimumab vs. Sorafenib	OS	OS	11 October 2017	27 August 2026
NCT02576509 (CHECKMATE459)	PHASE3	Systemic	Nivolumab vs. Sorafenib	OS	ORR	7 December 2015	7 February 2024
NCT03412773 (RATIONALE-301)	PHASE3	Systemic	Tislelizumab vs. Sorafenib	OS	ORR	28 December 2017	14 December 2023

The table summarizes clinical trials of immune checkpoint inhibitor (ICI) therapy for patients with unresectable hepatocellular carcinoma who received first-line ICI therapy, alone or in combination with tyrosine kinase inhibitors. Abbreviations: OS, Overall Survival; PFS, Progression-Free Survival; RECIST, Response Evaluation Criteria in Solid Tumors; PFS-IRF, Independent Review Facility-Assessment; BICR, Blinded Independent Central Review; ORR, Objective Response Rate. * Only one secondary outcome measure is presented for brevity. Additional secondary outcome measures were reported in the original trial publication.

## Data Availability

Data available in a publicly accessible repository.

## Abbreviations

The following abbreviations are used in this manuscript:

AASLD	American Association for the Study of Liver Diseases
AE(s)	Adverse event(s)
AFP	Alpha-fetoprotein
APASL	Asia-Pacific Association for the Study of the Liver
BCLC	Barcelona Clinic Liver Cancer
CASL	Canadian Association for the Study of the Liver
CD3	Cluster of Differentiation 3
CD8	Cluster of Differentiation 8
CI	Confidence interval
CNLC	Chinese Liver Cancer
CT	Computed tomography
CTC(s)	Circulating tumor cell(s)
CTLA-4	Cytotoxic T-lymphocyte–associated protein 4
DFS	Disease-free survival
EASL	European Association for the Study of the Liver
EFS	Event-free survival
EMA	European Medicines Agency
EV(s)	Extracellular vesicle(s)
FDA	U.S. Food and Drug Administration
GPC3	Glypican-3
HBV	Hepatitis B virus
HCC	Hepatocellular carcinoma
HCV	Hepatitis C virus
HKLC	Hong Kong Liver Cancer
HR	Hazard ratio
ICI(s)	Immune checkpoint inhibitor(s)
ITA.LI.CA	Italian Liver Cancer
LAG-3	Lymphocyte activation gene-3
LT	Liver transplant/transplantation
MAFLD	Metabolic dysfunction-associated fatty liver disease
MASH	Metabolic dysfunction-associated steatohepatitis
MASLD	Metabolic dysfunction-associated steatosis liver disease
MPR	Major pathological response
MRI	Magnetic resonance imaging
MSI	Microsatellite instability
MVI	Microvascular invasion
NGS	Next-generation sequencing
ORR	Objective response rate
OS	Overall survival
PD-1	Programmed cell death protein 1
PD-L1	Programmed death-ligand 1
PFS	Progression-free survival
PNPLA3	Patatin-like phospholipase domain-containing protein 3
QoL	Quality of life
R0	Microscopically margin-negative resection
RECIST	Response Evaluation Criteria in Solid Tumors
RFA	Radiofrequency ablation
RFS	Recurrence-free survival
SBRT	Stereotactic body radiotherapy
SOP(s)	Standard operating procedure(s)
TACE	Transarterial chemoembolization
TCR	T-cell receptor
TIL(s)	Tumor-infiltrating lymphocyte(s)
TKI(s)	Tyrosine kinase inhibitor(s)
TME	Tumor microenvironment
TTR	Time to recurrence
UCSF	University of California, San Francisco
VEGF	Vascular endothelial growth factor
VEGFR(s)	Vascular endothelial growth factor receptor(s)
c-KIT	Tyrosine-protein kinase Kit
ctDNA	Circulating tumor DNA
irAE(s)	Immune-related adverse event(s)
mOS	Median overall survival
mRECIST	Modified Response Evaluation Criteria in Solid Tumors
miRNA(s)	MicroRNA(s)
pCR	Pathological complete response
